# NcRNA: key and potential in hearing loss

**DOI:** 10.3389/fnins.2023.1333131

**Published:** 2024-01-17

**Authors:** Keyu Zhu, Ting Wang, Sicheng Li, Zeming Liu, Yuanyuan Zhan, Qi Zhang

**Affiliations:** ^1^Department of Plastic and Cosmetic Surgery, Tongji Hospital, Tongji Medical College, Huazhong University of Science and Technology, Wuhan, China; ^2^Department of Medical Ultrasound, Tongji Hospital of Tongji Medical College of Huazhong University of Science and Technology, Wuhan, China; ^3^Department of Plastic Surgery, Renmin Hospital of Wuhan University, Wuhan, Hubei, China

**Keywords:** hearing loss, non-coding RNA, micro-RNA, long-stranded non-coding RNA, sensorineural hearing loss, age-related hearing loss

## Abstract

Hearing loss has an extremely high prevalence worldwide and brings incredible economic and social burdens. Mechanisms such as epigenetics are profoundly involved in the initiation and progression of hearing loss and potentially yield definite strategies for hearing loss treatment. Non-coding genes occupy 97% of the human genome, and their transcripts, non-coding RNAs (ncRNAs), are widely participated in regulating various physiological and pathological situations. NcRNAs, mainly including micro-RNAs (miRNAs), long-stranded non-coding RNAs (lncRNAs), and circular RNAs (circRNAs), are involved in the regulation of cell metabolism and cell death by modulating gene expression and protein-protein interactions, thus impacting the occurrence and prognosis of hearing loss. This review provides a detailed overview of ncRNAs, especially miRNAs and lncRNAs, in the pathogenesis of hearing loss. We also discuss the shortcomings and issues that need to be addressed in the study of hearing loss ncRNAs in the hope of providing viable therapeutic strategies for the precise treatment of hearing loss.

## 1 Introduction

Based on the Global Burden of Disease (GBD) study, deafness is the third major cause of disability in the world, which affects more than 5% of the population overworld (Tyrovolas et al., 2022). Meanwhile, more people are suffering from mild, undiagnosed hearing loss. About 1.57 billion of the world population were impacted by hearing loss, a number that could grow to 2.45 billion by 2050 (Haile et al., 2021). Thus, hearing loss is an emerging serious problem. The World Health Organization (WHO) classifies hearing loss as mild, moderate, severe, and more severe, corresponding to levels of 26–40, 41–60, 61–80, and 81 dB HL or higher (Nieman and Oh, 2020). Long-term hearing loss may negatively impact life qualities. Communication barriers, social isolation, and complex mental health issues, are potentially some of the serious consequences of hearing loss (Lin et al., 2013; Mener et al., 2013; Bainbridge and Wallhagen, 2014; Mick et al., 2014; Kamil and Lin, 2015).

According to the location of the lesion, hearing loss could be categorized as conductive hearing loss, sensorineural hearing loss (SNHL), and mixed hearing loss (Shapiro et al., 2021). Obstructions and diseases in the outer or middle ear could prevent the normal transmission of sound from the environment to the eardrum and middle ear, leading to conductive hearing loss (Hill-Feltham et al., 2021). For adults, earwax impaction, otosclerosis, otitis media, and secretory otitis media are the most common causes of conductive hearing loss. In children, the most frequent causes are middle ear effusion and congenital malformations of the ear canal (Michels et al., 2019). Sensorineural hearing loss is caused by dysfunction in the cochlea and spiral ganglion or lesions in the auditory center. Age-related hearing loss (AHL), chronic noise-induced hearing loss (NIHL), and ototoxic drug-related hearing loss, are the major types of sensorineural hearing loss (Wang et al., 2019; Prince and Stucken, 2021). AHL is a bilateral, symmetrical, progressive pathologic process in which the extent of hearing loss increases with age (Pacala and Yueh, 2012; Cunningham and Tucci, 2017). NIHL often occurs with AHL and is difficult to distinguish, the patients usually have a significant history of noise exposure (Sliwinska-Kowalska and Davis, 2012; Chen K. et al., 2020). However, if the ears are exposed to asymmetrical noise, hearing loss could also occur asymmetrically. Hearing loss induced by ototoxic drugs usually develops with injury of hair cells, vascular striae, or the auditory nerve. Typical ototoxic drugs include salicylic acid preparations, cisplatin, and aminoglycoside antibiotics, as well as some diuretics (Li and Chai, 2019; Dillard et al., 2021). Although many factors participate in the development and progression of hearing loss, genetics may be the most common causative factor (Cai et al., 2017; Mittal et al., 2017; Azadegan-Dehkordi et al., 2018).

**FIGURE 1:**
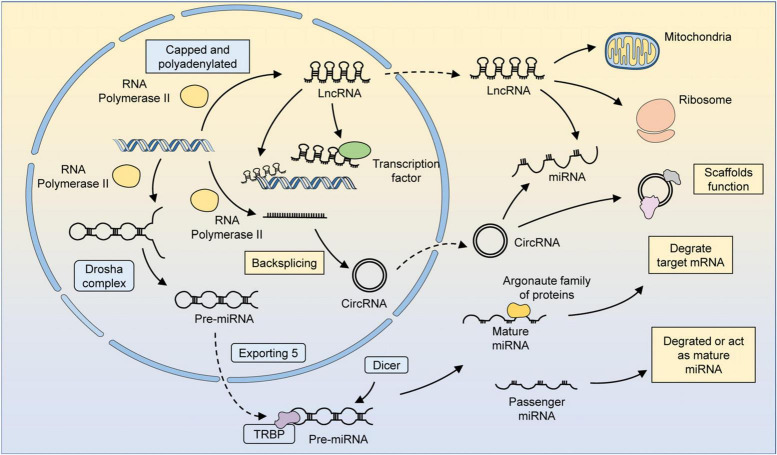
The biogenesis and function of several major ncRNAs. MiRNAs are transcribed by RNA polymerase 2/3 to form primary miRNAs (pri-miRNAs). Pri-miRNAs have specific hairpin loops that can be recognized and cleaved by the Drosha complex along the RNA-binding protein DGCR8. The cleaved pri-miRNA forms a precursor miRNA, which then translocates from the nucleus to the cytoplasm via exportin 5. Precursor miRNAs can be processed by Dicer in an RNA-binding protein TRBP-dependent manner to form double-stranded somatic RNAs. One strand of the double-stranded somatic RNA is loaded into the RNA-induced silencing complex (RISC) as a mature miRNA, leading to the degradation of target mRNAs. The other strand, known as a transient miRNA, is usually degraded. However, in some cases, these miRNAs can also function as mature miRNAs. LncRNAs could be transcription products of different DNA elements, such as promoters, enhancers, and exons. Most lncRNAs are transcribed by RNA polymerase 2 and have similar characteristics as mRNAs, but lncRNAs do not possess translatable ORFs. The regulatory mechanism of lncRNAs can be summarized in four ways: signaling, decoying, guiding, and scaffolding. LncRNAs localized in the nucleus can recruit factors for chromatin remodeling or directly bind to transcription factors and thus participate in chromatin modifications and epigenetic modifications. LncRNAs can be translocated by a mechanism similar to that of mRNAs, and those localized in the cytoplasm can regulate metabolism and organelle function by binding to organelles, such as mitochondria. LncRNAs can also bind to miRNAs or ribosomes to participate in the regulation of gene expression at the post-transcriptional level. CircRNAs are formed by reverse splicing of RNA polymerase 2 transcription products. CircRNAs can serve as scaffolds to facilitate the interaction of related proteins or participate in regulating the miRNA-mediated degradation of mRNAs.

The results of genome sequencing have proven that only 2–3% of the human genome is responsible for protein coding (Djebali et al., 2012). The coding genes, occupying a quite small portion of the genome, could be transcribed into mRNA and then translated and modified in three dimensions to form functional proteins. Non-coding RNAs (ncRNAs) are the major component of RNA, though not involved in coding proteins in general, these transcription products make up more than 90% of all RNA (Kopp and Mendell, 2018; Ransohoff et al., 2018). At first, ncRNAs are considered to be just massive junk sequences without protein-coding ability. The importance of ncRNAs in normal life regulation processes and disease processes has been gradually recognized, and the possibility of clinical applications has been explored (Adams et al., 2017; Anastasiadou et al., 2018). Micro-RNAs (miRNAs), long-stranded non-coding RNAs (lncRNAs), and circular RNAs (circRNAs) were the major components of regulatory ncRNAs (Adams et al., 2017; Zhu et al., 2018; Figure 1). These ncRNAs are widely engaged in the regulation of various life processes, including cell metabolism, development, proliferation, transcription, and post-transcriptional modifications (Deveson et al., 2017; Esposito et al., 2019).

Human cochlear tissue that could be used for studies is difficult to obtain, which limits the progress in the hearing loss area. The construction of applicable animal models and the development of new analytical techniques have improved this situation in recent years. Although ncRNA research is still lagging behind other fields, much relevant literature suggests that ncRNAs are equally relevant in the field of hearing loss. The study of these regulators could reveal the pathologic mechanisms of the relevant diseases, provide potential medicinal targets, and guide the development of therapeutic agents (van Zandwijk et al., 2017; Seto et al., 2018; Kimura, 2020).

In this review, we detailed the role of ncRNAs in hearing loss and their involvement in potential treatment options. We also discuss the current limitations and perspectives of this field, with the hope that more valuable discoveries will be made in the future of hearing loss research.

## 2 MiR-183 family in hearing loss

The MiR-183 family is a group of three miRNAs with conserved expression, including miR-183, miR-182, and miR-96 (Dambal et al., 2015; Mahmoodian Sani et al., 2016). They were first found to be abundantly expressed in hair cells and ear neurons in mice and zebrafish, and shared nearly identical sequences in humans. The specific expression and conserved sequences indicated their importance in auditory development and function, and they were also the most studied miRNAs in hearing loss.

Aberrant expression of the miR-183 cluster could induce hearing loss. Weston et al. (2006) confirmed that the miR-183 cluster was abundantly expressed in inner ear sensory neurons and hair cells of adult mice. In contrast, the expression of miR-181 family and miR-183 family was significantly down-regulated in the Corti organ of aging individuals, and these changes could be detected even before the onset of organ morphological changes and hearing loss (Zhang et al., 2013). Emphasizing their importance in hair cell differentiation and maturation. On the other hand, different kinds of aberrant expression of miR-183 clusters might lead to abnormal development and auditory functions of the inner ear. Overexpression of the miR-183 cluster could lead to progressive sensorineural hearing loss in mice (Weston et al., 2018). Li et al. (2010) found that duplicated ear cysts, ectopic or dilated sensory patches, extra hair cells, and morphologic abnormalities of the static auditory ganglion (SAG) occurred in a miR-96- or miR-182-overexpressed zebrafish model, while knocking down miR-183 gene cluster led to reduced inner ear hair cell numbers, smaller SAGs, hemianopsia defects, and posterior lateral line neuroma abnormalities. In mice, the inactivation of miR-183 gene cluster significantly affected the development, morphology, and function of cochlear hair cells, leading to severe hearing loss (Geng et al., 2018).

MiR-96 was the first miRNA to be identified concerning deafness. Mencia et al. first described two different point mutations in the seed region of miR-96, and the mutation found in two Spanish families caused non-syndromic progressive sensorineural hearing loss (Lewis et al., 2009; Mencía et al., 2009). The involvement of miR-96 in the pathogenesis of human deafness was further demonstrated by Soldà et al. (2012) who performed a genetic screen of 882 non-syndromic sensorineural hearing loss patients and 836 normal-hearing controls. They identified a putative novel mutation in miR-96 that reconstructed the secondary structure of the pre-miR-96 hairpin, which significantly affected the ability of mature miR-96 to regulate selected targets. In particular, mutations in miR-96 were found to cause more severe hearing loss in mice than mutations in the other two miRs, possibly due to the creation of new target genes and the loss of normally acting target genes (Lewis et al., 2020). Kuhn et al. (2011) further demonstrated that miR-96 mutations could lead to a pre-termination of sensory hair cell maturation in mice, as well as disrupted maturation of hair cell stereocilia bundles and disorganized remodeling of auditory nerve connections within the cochlea. These indicated that miR-96 profoundly influenced the process of cochlear hair cell physiology and their morphological differentiation. Although several genes, including Clic 5, Aqp 5, Odf, Celsr 2, Ryk, Myrip, and Ptprq, have been identified as possible targets of miR-96, their regulatory roles in the inner ear remain poorly understood (Lewis et al., 2009; Gu et al., 2013; Chen et al., 2014).

By transfecting miR-182, bone marrow stem cells (BMSCs) could be induced to express mRNAs for hair cell markers such as SOX2, POU4F3, and ATOH1, as well as ATOH1 protein, thus playing a key role in the differentiation of hair cell (Mahmoudian-Sani et al., 2018). Forkhead box O3a (FOXO3a) is a transcription factor of the FOXO family, which is associated with a variety of biological processes, such as cell cycle, apoptosis, and metabolism (Habrowska-Górczyńska et al., 2021). Li et al. (2016) found that the overexpressed miR-182 could inhibit apoptosis by suppressing the translation of FOXO3a, thus providing a protective effect on cisplatin-treated hair cells *in vitro*. Also, miR-182 increased the level of phosphatidylinositol-3 kinase (PI3K) regulatory subunit p85α in outer hair cells, consequently reducing the loss of hair cells and relieving steric cilia in the drug-associated hearing loss rat model induced by kanamycin and furosemide, leading to the mitigation of perpetual threshold shifts (Chen J. et al., 2020). These demonstrated the protective potential of miR-182 in drug-induced hearing loss. Transmembrane protease serine 3 (TMPRSS3) is a gene expressed in the fetal inner ear, and its mutation is associated with non-syndromic hereditary hearing loss (Guipponi et al., 2002). TMPRSS3 could regulate the apoptosis and survival of HEI-OC1 cells through the circ-Slc41a2-miR-182-Akt cascade, thereby acting a role in preventing hearing loss (Zhang Z. et al., 2019).

MiR-183 is aberrantly expressed in hearing loss at a high frequency. After exposure to white band noise, the corti organ, outer hair cells, and ABR threshold movement of rats could be impaired (Park S. et al., 2020). The miR-411-3p, miR-183-5p, miR-377-3p, miR-20b-5p, and miR-200b-3 in the cochlear nucleus were significantly altered, andmiR-92a-1-5p, miR-136-3p, and miR-26b-5p in the inferior colliculus also experienced significant changes. MiR-183 is also differentially expressed in idiopathic sudden sensorineural hearing loss (ISSNHL). Ha et al. (2020) found that miR-183, miR-210, miR-18b, and miR-23a were significantly differentially expressed in ISSNHL patients, and the sensitivity and specificity of their diagnostic efficacy was 80.95% (17/21) and 87.50% (21/24), respectively. This indicated that miR-183 might play an important role in the pathology of hearing loss. Further, Patel et al. (2013) found that Taok1 may be a target gene of miR-183 in NIHL using target prediction analysis. They validated the correlation *in vitro* model and concluded that the miR-183/Taok 1 target pair might participate in regulating the cochlear degenerative process after sound overstimulation (Patel et al., 2013). The impact of miR-183/miR-96 on normal synaptic transmission and the development of the auditory hindbrain was probably another mechanism that contributed to the development of hearing loss (Krohs et al., 2021). In hair cells, inhibition of Notch signaling promoted hair cell differentiation and regeneration, and miR-183 inhibition abolished this promoting effect, thus demonstrating the important involvement of miR-183 in hair cell differentiation and regeneration (Zhou et al., 2018).

## 3 MiR-34a in hearing loss

miR-34a, a member of the miR-34 family, is first described as a tumor suppressor miRNA that can be expressed induced by p53 (Raucci et al., 2021). With intensive investigation, the role of miR-34a has been revealed in a variety of life processes, both in neurogenesis and differentiation and in cancer progression (Chua and Tang, 2019).

Li et al. (2017) sequenced samples from SSNHL patients and normal individuals to detect differentially expressed miRNAs, and further constructed a miRNA-target-protein-protein interaction (PPI) network by combining the obtained results with database (Zhou H. et al., 2023). Hsa-miR-34a/15a/23a/210/18b/548n/143 had the most target genes in the miRNA-target-PPI network, suggesting their extensively involvement in the pathogenesis of SSNHL. MiR-34a levels in the cochlea, auditory cortex, and plasma of mice were elevated during aging, while sirtuin-1 (SIRT1), Bcl-2, and E2F3 levels were decreased with increasing age (Pang et al., 2016). In AHL, miR-34a overexpression could promote apoptosis by inhibiting Bcl-2, as well as by inhibiting SIRT1 and increasing HIF-1α and the acetylation of p53, thus contributing to the progression of hearing loss (Xiong et al., 2015; Huang et al., 2017). In HEI-OC1 cells, the overexpression of miR-34a could regulate autophagy and biogenesis of mitochondrial by suppressing SIRT1, and also lead to a reduction of ATG9A and impaired autophagic processes, while rapamycin could protect HEI-OC1 cells by restoring autophagic fluxes (Lin et al., 2017; Pang et al., 2017; Xiong et al., 2019).

**FIGURE 2:**
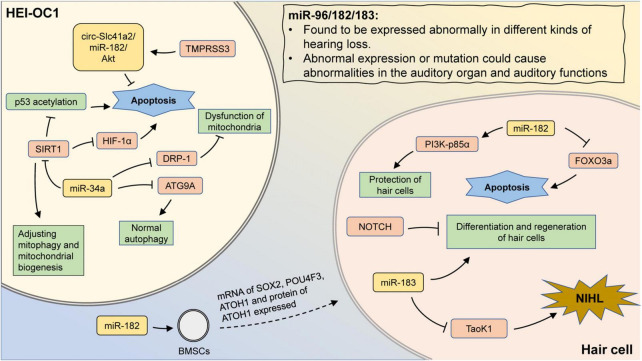
The mechanism involving miR-183 family and miR-34a in hearing loss. MiR-96/182/183 are found to be expressed abnormally in different kinds of hearing loss. Also, aberrant expression or mutation could cause abnormalities in the auditory organ and auditory functions. Transfection of miR-182 promotes the expression of hair cell-related markers in BMSCs. In hair cells, miR-182 can inhibit apoptosis by suppressing FOXO3a, as well as protect hair cells by promoting PI3K-p85α expression. Inhibition of Notch signaling promotes hair cell proliferation and differentiation, which could be abolished by miR-183 inhibition. In addition, aberrant expression of miR-183 may attenuate the inhibition of Taok1 and contribute to the development of NIHL. In HEI-OC1 cells, inhibition of DRP-1 by miR-34a can lead to mitochondrial dysfunction. MiR-34a can also regulate mitophagy and mitochondrial biogenesis by suppressing SIRT1. The inhibition of SIRT1 could promote the p53 acetylation and reduce the expression of HIF-1α, leading to cell apoptosis. MiR-34a inhibits ATG9A and lead to impaired autophagy. TMPRSS3 inhibits apoptosis through the circ-Slc41a2/miR-182/Akt pathway.

In other types of hearing loss, aberrant expression of miR-34a was also involved in disease progression. The overexpression of miR-34a inhibited DRP-1 expression and led to mitochondrial dysfunction as well as exacerbation of cisplatin-induced ototoxicity (Wang et al., 2023). In the cochlea of db/db mice with diabetes mellitus, miR-34a was found to be significantly upregulated and accompanied by significant hearing threshold elevation and hair cell loss, implying that miR-34a could serve as a potential therapeutic target for diabetes-related hearing loss (Lin et al., 2017; Figure 2).

## 4 Other miRNAs in hearing loss

### 4.1 miRNAs in age-related hearing loss

Zhang J. et al. (2019) identified 799 differentially expressed genes in the cochlear tissue of Cmah-deficient mice, and found that the down-regulated differentially expressed genes were mainly involved in the PPAR signaling pathway. Among them, mmu-miR-130b-3p, mmu-miR-27a-3p, mmu-miR-27b-3p, and mmu-miR-721 were predicted to regulate PPARG, which may be an important mechanism in AHL. While in the miRNAs that appeared to be differentially expressed in the lateral wall of the cochlear duct in senescent mice, most of the down-regulated miRNAs were known pathways that regulate cell proliferation and differentiation, while all of the up-regulated miRNAs were associated with apoptosis (Zhang et al., 2014). MiR-29b, a miRNA significantly up-regulated in cochlear hair cells of senescent mice, was able to inhibit the expression of SIRT1 and PGC-1α in HEI-OC1 cells, leading to mitochondrial dysfunction and increased apoptosis. While MIAT could protect HEI-OC1 cells through down-regulating miR-29b (Xue et al., 2016; Hao et al., 2019). MiR-34a and lncRNA H19 were also involved in the SIRT1-mediated regulation of cytoprotective functions, indicating a possible synergistic role of specific ncRNAs in the development of hearing loss.

### 4.2 miRNAs in noise-induced hearing loss

Noise is a common cause of hearing loss, with an estimated 1.3 billion people worldwide suffering from hearing loss due to noise exposure (Vos et al., 2012). NIHL is a complex disease with a combination of genetic and environmental factors, so susceptibility may vary widely between individuals. Several studies have revealed that ncRNAs may be part of the pathogenesis of noise-induced hearing loss.

Miao et al. (2021) investigated the relevance of SNPs in the AKT2 gene to NIHL. They demonstrated that the SNP rs2304186 in the AKT2 3′-UTR could alter the binding affinity of hsa-miR-625-5p to the mutant region in an allele-specific manner, by which the susceptibility to NIHL might be changed. KCNQ4 is a voltage-gated potassium channel, and is critical for hearing preservation due to the capability of maintaining ionic homeostasis in hair cells. Wang et al. (2021) found that KCNQ4 levels were markedly decreased by miR-153, which was significantly increased in mice that developed sensory neural hearing loss after noise exposure. Knockdown of miR-153 could increase KCNQ4 levels and relieve impaired hearing (Wang et al., 2021). Another miRNA, miR-1229-5p, was notably elevated in the serum of patients with occupational noise-induced hearing loss (ONIHL), and its overexpression might take part in the pathogenesis of ONIHL by inhibiting MAPK1 signaling (Li et al., 2018). In addition, miR-185-5p and miR-451a had elevated levels in the plasma of NIHL patients, hoping to serve as biomarkers for NIHL (Ding et al., 2016).

### 4.3 miRNAs in drug-associated hearing loss

Alterations in miRNA expression profiles in the inner ear following ototoxic drugs could contribute to understand the mechanisms by which these ototoxic drugs cause hearing loss. These altered markers also help to identify potential markers for diagnostic and therapeutic applications. In addition to the well-known miR-183 cluster, several miRs have also been identified that may play a role in drug-induced hearing loss.

Cisplatin is widely used in the chemotherapy of tumors, and is also commonly used in combination with other drugs or alone in the treatment protocols of multiple cancers (Tang et al., 2021). However, cisplatin could cause several side effects, including ototoxicity, nephrotoxicity, and neurological damage. Regulation of differentially expressed ncRNAs may be an effective way to minimize the toxic side effects of cisplatin. Liu et al. constructed a hearing loss model by intraperitoneally injecting cisplatin into mice, attempting to explore the protective effect of resveratrol against cisplatin-induced hearing loss (Liu et al., 2021). They found that miR-455-5p could be upregulated by resveratrol, thereby activating the PTEN-PI3K-Akt signaling pathway and attenuating the ototoxic effect of cisplatin. Forkhead box G1 (FOXG1) was a member of the FOX transcription factor gene family and exerted an important role in neuronal cell development and cell cycle regulation (Tigani et al., 2020). Cisplatin treatment decreased FOXG1 expression in mice and reduced cellular autophagy through its downstream target miRNAs, leading to the accumulation of reactive oxygen species (ROS) and cochlear hair cell death (Mu et al., 2023). Mu et al. (2023) rescued the reduced autophagy and relieved the ear injury caused by cisplatin by overexpressing FOXG1 and its target miRNA miR-34 and miR-183 family. In addition, Tsai et al. (2021) demonstrated that exosome injection may exert a protective effect against drug-induced hearing loss through miRNAs. After exosomal injection with umbilical cord MSCs, the mRNA levels of SMN1 and Pona were significantly up-regulated in cisplatin-treated mice, while the expression levels of mmu-miR-125b-5p, mmu-miR-125a-5p, and mmu-miR-127-3p in the inner ear tissues were also markedly improved, thus yielding a significant rescue of hearing loss in the mice.

Some antibiotics are ototoxic, principally including streptomycin and aminoglycoside antibiotics (Kros and Steyger, 2019). Lee et al. (2018) treated mice with kanamycin and furosemide to disrupt their hearing, showing that the chronological order of miR-205 elevation in the inner ear was consistent with the order of functional impairment occurring in the inner ear. This demonstrates that miR-205 was directly engaged in the process of ototoxicity and could be used as a potential diagnostic molecular marker. Ding and Wang (2022) also constructed a hearing loss model by using gentamicin in rats, demonstrating that miR-106a could promote oxidative stress-induced SNHL by targeting connexin-43.

In conclusion, several preclinical studies have confirmed the involvement of miRNAs in the process of inner ear injury in drug-induced hearing loss. Platinum drugs and some antibiotics are the most common drugs with ototoxicity, as targeted modulation of some miRNAs performed in rodent models effectively mitigated the hearing loss induced by them, indicating that miRNAs have the potential to be therapeutic targets for Drug-Induced Hearing Loss.

### 4.4 miRNAs in sudden sensorineural hearing loss

Sudden sensorineural hearing loss (SSNHL) has no identifiable cause and is characterized by a hearing loss of ≥30 dB HL in at least three consecutive frequencies (Chandrasekhar et al., 2019). Treatment response in SSNHL varies widely between individuals, and seriously impacts the quality of patient survival (Kang et al., 2020; Park K. et al., 2020). Exploring the pathogenesis of SSNHL is essential for the development of novel prognoses and therapeutic options. SSNHL is associated with vasodilatation, infection, and immune abnormalities, yet its pathogenesis is poorly understood. (Yamada et al., 2022). In the absence of an accurate causative factor, it was difficult to simulate a disease model that reflects the pathological state of SSNHL, which further limits the advancement of this field.

Several studies suggested that miRNAs might be involved in SSNHL pathogenesis. Dicer and Drosha are two RNase III proteins that play an important role in miRNA biogenesis and have been shown to engage in a variety of diseases (Ha and Kim, 2014). Kim et al. examined the mRNA level of Dicer and Drosha in the blood of 57 SSNHL patients and 50 healthy volunteers, and found that dicer mRNA was significantly down-regulated in SSNHL patients, while no obvious change was observed in drosha mRNA (Kim et al., 2015). Similarly, AGO2, another component of the miRNA biogenesis process was found to be expressed differentially in SSNHL (Han et al., 2014). These aberrantly expressed proteins influenced miRNA processes. Although a causal relationship between these processes and SSNHL had not been confirmed, this indicated the presence of miRNA abnormalities in SSNHL. Abnormally expressed miRNAs were also present in exosomes from peripheral blood in SSNHL patients (Zhang et al., 2023b). Among them, PC-5p-38556_39, PC-5p-29163_54, and miR-93-3p showed remarkable differential expression, which might be closely related to the pathogenesis of SSNHL. In addition, the expression levels of serum miR-195-5p/-132-3p/-30a-3p/-128-3p/-140-3p/-186-5p/-375-3p/-590-5p showed a difference after the onset of sudden deafness, and this difference remained unchanged for a year thereafter (Nunez et al., 2020; Abgoon et al., 2023).

The specific role of these miRNAs in the pathogenesis of SSNHL is unknown, but has been explored with some application. Shew et al. (2019) built a machine-learning disease-specific algorithm that was able to predict the existence of sensorineural hearing loss by using the miRNA expression profiles of ectopic lymphoid lymphocytes. This potentially represents a trend in the future of disease diagnosis (Shew et al., 2019).

### 4.5 miRNAs in hair cell regeneration

Hair cells are a key factor in auditory conduction, as they could convert mechanical acoustic stimuli into electrochemical signals, allowing the transmission of hearing via the auditory nerve to the auditory center. Since the hair cells of mammals are non-renewable, a variety of genetic alterations and environmental factors that damage the hair cells are capable of causing irreparable hearing loss (Burns and Corwin, 2013). To date, we have a limited understanding of the mechanisms involved in the repair and regeneration of hair cells. Several combinations of growth factors and modulation of intracellular signaling pathways have been used to for testing the capacity to induce hair cell regeneration with fine results (Lambert, 1994; Yamashita and Oesterle, 1995; Zheng and Gao, 2000; Huang et al., 2009; Wu et al., 2016; Qian et al., 2021). However, these regenerated hair cells were unable to fully recover their previous quantity, while they were also lacking the normal functions of mature hair cells. Promoting the regeneration of completely functional hair cells is critical for the recovery of hearing. MiR-125 inhibited the proliferation of cochlear progenitor cell CPCs by down-regulating CDK2, compromising the regeneration of hair cells (Peng et al., 2021). LIN28B enhanced the plasticity of naive supporting cells in a mTORC1-dependent manner, thereby inducing the generation of hair cells, while this effect could be antagonized by miR-let-7g overexpression (Li and Doetzlhofer, 2020). MiR-210 was another novel factor that could promote hair cell generation (Riccardi et al., 2016). Riccardi et al. (2016) used next-generation miRNA sequencing to identify the most prominently expressed miRNAs in a mouse inner ear cell line UB/OC-1 during its differentiation to a hair cell phenotype, and validated the function of these miRNAs *in vitro*. They revealed that miR-210 could increase the production of new hair cells by promoting the transdifferentiation of supporting epithelial cells, suggesting that miR-210 was a potential therapy for hearing loss (Table 1).

**TABLE 1:** NcRNAs and their roles in hearing loss.

NcRNAs	Targeted genes/proteins	Impact on hearing loss	Reference
miR-96	Possibly Clic 5, Aqp 5, Odf, Celsr 2, Ryk, Myrip, and Ptprq	Promote the development and maturation of hair cells, affect the remodeling of auditory nerve connections	Krohs et al., 2021; Lewis et al., 2020; Lewis et al., 2009
miR-182	FOXO3a, PI3Kp85α,Akt	Impact apoptosis and promote hair cell development and differentiation	Li et al., 2017; Li et al., 2022; Li et al., 2016
miR-183	Taok1, Notch	Participate in cochlear degenerative processes and promote regeneration and differentiation of hair cells	Lin et al., 2017; Mahmoodian Sani et al., 2016
miR-34a	Bcl-2, SIRT1, HIF-1α, p53, ATG9A, DRP-1	Regulate apoptosis, autophagy and mitochondrial function	Miao et al., 2021; Michels et al., 2019; Mick et al., 2014; Mittal et al., 2017; Mu et al., 2023; Nieman and Oh, 2020; Nunez et al., 2020
miR-29b	SIRT1, PGC-1α	Increase apoptosis and mitochondrial dysfunction	Pang et al., 2016; Park K. et al., 2020
miR-153	KCNQ4	Disrupt the ionic balance in hair cells	Peng et al., 2021
miR-1229-5p	MAPK1	Engage in the pathogenesis of ONIHL	Prince and Stucken, 2021
miR-455-5p	PTEN-PI3K-Akt,	Alleviate the ototoxic effects of cisplatin	Ransohoff et al., 2018
miR-106a	connexin-43	Promote oxidative stress-induced SNHL	Shew et al., 2019
MiR-125	CDK2	Inhibit the proliferation of cochlear progenitor cells	Wang et al., 2021
miR-let-7g	LIN28B	inhibit the regeneration of hair cells	Wang and Sun, 2022
miR-210	BDNF, Hoxa 1, Kctd 11, Dtx 1	Promote the regeneration and differentiation of hair cells	Weston et al., 2006

## 5 LncRNA in hearing loss

LncRNA is a class of ncRNA longer than 200 bp, and is processed similarly to protein-coding genes, and has cell- and tissue-specific expression patterns (Quinn and Chang, 2016). LncRNAs are associated with a variety of diseases, including tumors, cardiovascular diseases, and inflammatory diseases. The role and mechanism of lncRNAs in hearing loss have been relatively poorly reported, compared to studies of miRNAs

With the analysis of the co-expression profiles of mRNAs and lncRNAs in the ARHL-associated RNA sequencing dataset, Kim et al. (2023) identified 112 mRNAs and 10 lncRNAs with relatively high expression in the cochlea of aged mice. Wang et al. (2017) also demonstrated that in the Chinese population, the polymorphisms in LncRNA HOTAIR also could affect the risk of NIHL. This suggested that lncRNA was involved in hearing loss. In aged mice, a total of 738 lncRNAs and 2033 mRNAs were found to be differentially expressed, with lncRNA NONMMUT010961.2 being the most significantly differentially expressed lncRNA (Zhao et al., 2020). The knockdown of lncRNA NONMMUT010961.2 reduced the expression levels of oxidative stress-related gene Ar and hearing loss-related gene Hgf in HEI-OC1 cells, proving the participation of lncRNA NONMMUT010961.2 in the pathogenesis of AHL. Oxidative stress injury associated with increasing age was a critical mechanism of age-related hearing loss, and several lncRNAs had been found to act in regulating oxidative stress in cells. LncRNA H19 was found to be notably down-regulated in the cochlea of aged mice, and could inhibit the oxidative stress injury in cochlear hair cells through miR-653-5p/SIRT1 axis (Xie et al., 2022). Li et al. (2022) demonstrated that lncRNA Gm44593 could regulate the miR-29b/WNK axis to improve oxidative stress injury in HEI-OC1 cells. MiR-204-5p reduced the viability of spiral ganglion neurons (SGNs), which was achieved by inhibiting the expression of the TMPRSS3. In addition, the Hdac2/Sp1/miR-204-5p/Bcl-2 regulatory axis was also implicated in the apoptotic process of cochlear cells in acute hearing loss (Jiang et al., 2020; Xie et al., 2021). Jiang et al. (2020) found that lncRNA EBLN3P could competitively inhibit the effect of miR-204-5p and regulate the expression of TMPRSS3, thus effectively promoting the restoration of impaired SGN function. This revealed a key regulatory role for lncRNAs as new therapeutic targets in hearing loss.

Immune abnormalities are a major cause of hearing loss. Before 1979, the inner ear was considered an immunologically exempt organ, making it rare for researchers to explore the role of immunologically aberrant mechanisms in the development of hearing loss. The reports of McCabe (1979) and Rask-Andersen and Stahle (1979) brought to light that the inner ear was not only in contact with the host immune system, but was also susceptible to immune disorders. Zhang et al. (2023a) constructed a mouse model of immune-associated hearing loss by injecting inner ear antigens, and analyzed the plasma exosomes to identify a total of differentially expressed 94 lncRNAs, 612 mRNAs, and 100 miRNAs. Through analysis and screening, a ceRNA regulatory network was constructed based on 74 lncRNAs, 28 miRNAs, and 256 mRNAs. Among these, lncRNAs Gm9866 and Dusp7, were significantly up-regulated, while miR-185-5p was decreased, with an interaction between the three. This demonstrated the potential involvement and regulation of these three ncRNAs in hearing loss.

## 6 Discussion and prospect

There is an underlying genetic component in most causes of hearing loss, and identifying the involvement of coding and non-coding genes is critical to the study of hearing loss. Over the last decade, advances in sequencing technologies and assays have facilitated the identification of new genes and novel mutations associated with hearing loss. The importance of ncRNAs in the field of hearing loss is well established, although overall research progress still lags behind. A variety of miRNAs, represented by the miR183 family, are extensively involved in the genesis and regulation of hearing loss at various stages. However, there are still some areas of ncRNAs in hearing loss that deserve to be deepened.

Firstly, miRNA sequencing has identified hundreds of miRNAs expressed in the mouse inner ear, but the roles of most of them remain uncharacterized. The miR-183 family is most studied in hearing loss, but the regulatory networks are still imperfectly understood. For most of the miRNAs that are significantly differentially expressed in hearing loss, we have not fully elucidated the pathways involved and the related mechanisms in hearing loss. The study of miRNA mechanism in hearing loss would help us to identify the key miRNAs involved in hearing loss, and we can also try to explore the precursors that lead to the occurrence of these abnormalities.

Second, we emphasize here the role and mechanism of ncRNAs in hearing loss, but most of the ncRNAs involved are still predominantly miRNAs, while other lncRNAs and circRNAs have not been elucidated in depth. LncRNAs and circRNAs are the upstream regulatory units of miRNAs, and can regulate the hearing loss of ncRNAs by affecting miRNAs and regulating their corresponding proteins to fulfill their biological regulatory functions. These functions are indispensable for the occurrence and development of hearing loss. Therefore, the enrichment of potential ncRNAs in hearing loss through multi-omics and other sequencing tools is necessary for early diagnosis, effective intervention, and prognosis of hearing loss.

Thirdly, hair cells are a key component of the auditory system, and many causes of hearing loss cross over at the point of hair cell injury. There are already some treatments that target hair cells, such as promoting the conversion of support cells into hair cells, thus accomplishing the replenishment of injured hair cells. However, the induced-differentiated hair cells usually do not have fully normal function, but instead are in an intermediate phenotype between support cells and hair cells. An improved understanding of the mechanisms of hair cell damage is necessary to overcome these difficulties. Some miRNAs, such as miR-210, can serve as possible contributors to hair cell regeneration. Meanwhile, targeted delivery using ncRNAs has been performed in many clinical/pre-clinical trials, especially in the treatment of cancer. Combining ncRNAs with nanomaterials permits targeted delivery and controlled release of ncRNAs, which holds tantalizing prospects for the treatment of hearing loss (Dai et al., 2022; Wang and Sun, 2022; Zhou et al., 2022; Zhou L. et al., 2023). The development of ncRNA-targeted drugs for hearing loss treatment is also one of the main goals of related studies.

Finally, the difficulty of obtaining cochlear tissue has been hindering progress in ear disease research, as analyzed tissue samples are necessary to study disease models. Easier and quicker approaches are needed to obtain cochlear tissue samples that can be used for research. The construction of animal models is one option, but there is a lack of widely recognized disease models for hearing loss. In recent years, advances in analytical and detection techniques have allowed us to use smaller amounts of tissue for analysis, which greatly facilitated research related to hearing loss. However, this is still insufficient for the extremely valuable inner ear tissue of humans. On the other hand, using less tissue for analysis means the possibility of more unpredictable bias, thus how to prevent this from affecting the accuracy of the results needs to be considered.

In conclusion, the role of ncRNAs in hearing loss is becoming increasingly prominent. Specially, miRNAs are often found to be abnormally expressed or mutated in several types of hearing loss, including AHL, NIHL, drug-associated hearing loss, and SSNHL. The most common examples are miR-183 family and miR-34a, both of which can be involved in processes such as apoptosis, autophagy, and hair cell regeneration through downstream proteins and signaling pathways, thereby affecting the onset and progression of hearing loss. LncRNAs are also found to be widely expressed aberrantly in hearing loss, but the specific regulatory mechanisms associated with these aberrantly expressed lncRNAs remain to be further investigated. The excavation and mechanism exploration of ncRNAs will provide new insights into the pathogenesis of unheard hearing loss and novel therapeutic approaches.

## Author contributions

KZ: Writing – original draft. TW: Writing – original draft. SL: Writing – original draft. ZL: Writing – review and editing. YZ: Writing – review and editing. QZ: Writing – review and editing.
